# The changes of immunoglobulin G *N*-glycosylation in blood lipids and dyslipidaemia

**DOI:** 10.1186/s12967-018-1616-2

**Published:** 2018-08-29

**Authors:** Di Liu, Xi Chu, Hao Wang, Jing Dong, Si-Qi Ge, Zhong-Yao Zhao, Hong-Li Peng, Ming Sun, Li-Juan Wu, Man-Shu Song, Xiu-Hua Guo, Qun Meng, You-Xin Wang, Gordan Lauc, Wei Wang

**Affiliations:** 1 Beijing Key Laboratory of Clinical Epidemiology, School of Public Health, Capital Medical University, 10 Youanmen Xitoutiao, Beijing, 100069 China; 2 Center for Physical Examination, Xuanwu Hospital, Capital Medical University, Beijing, 100050 China; 3 School of Medical Sciences, Edith Cowan University, Perth, WA 6027 Australia; 4 Genos Glycobiology Research Laboratory, 10000 Zagreb, Croatia; 5 Faculty of Pharmacy and Biochemistry, University of Zagreb, 10000 Zagreb, Croatia

**Keywords:** Immunoglobulin G, *N*-Glycosylation, Blood lipids, Dyslipidaemia

## Abstract

**Background:**

Alternative *N*-glycosylation has significant structural and functional consequences on immunoglobulin G (IgG) and can affect immune responses, acting as a switch between pro- and anti-inflammatory IgG functionality. Studies have demonstrated that IgG *N*-glycosylation is associated with ageing, body mass index, type 2 diabetes and hypertension.

**Methods:**

Herein, we have demonstrated patterns of IgG glycosylation that are associated with blood lipids in a cross-sectional study including 598 Han Chinese aged 20–68 years. The IgG glycome composition was analysed by ultra-performance liquid chromatography.

**Results:**

Blood lipids were positively correlated with glycan peak GP6, whereas they were negatively correlated with GP18 (*P *< 0.05/57). The canonical correlation analysis indicated that initial *N*-glycan structures, including GP4, GP6, GP9-12, GP14, GP17, GP18 and GP23, were significantly correlated with blood lipids, including total cholesterol, total triglycerides (TG) and low-density lipoprotein (r = 0.390, *P* < 0.001). IgG glycans patterns were able to distinguish patients with dyslipidaemia from the controls, with an area under the curve of 0.692 (95% confidence interval 0.644–0.740).

**Conclusions:**

Our findings indicated that a possible association between blood lipids and the observed loss of galactose and sialic acid, as well as the addition of bisecting GlcNAcs, which might be related to the chronic inflammation accompanying with the development and procession of dyslipidaemia.

**Electronic supplementary material:**

The online version of this article (10.1186/s12967-018-1616-2) contains supplementary material, which is available to authorized users.

## Background

Glycosylation is an essential posttranslational modification of proteins. It is an integral part of proteins and significantly contributes to their structure and function [1–3]. Immunoglobulin G (IgG) plays an important role in the human immune system, and *N*-glycans attach to the conserved asparagine 297 in the fragment crystallizable (Fc) part of this molecule and act as a switch between pro- and anti-inflammatory IgG functionality [1, 4–6]. IgG *N*-glycosylation impacts the physiology and malfunction of the immune system, and aberrant IgG *N*-glycosylation is involved in several inflammatory and chronic diseases [7–10]. Previous studies have revealed that genetic loci associated with variation in IgG glycosylation are also known risk factors for several inflammatory diseases and chronic diseases [11, 12], indicating that IgG glycosylation is not merely a result of complicated enzymatic activities, but is a subtly regulated outcome designed to meet dominant physiological needs.

It is widely recognized that dyslipidaemia is associated with an increased risk of coronary artery disease, stroke, and heart failure [13–16]. However, the mechanisms by which dyslipidaemia increase the risk of these diseases has not been completely elucidated. Importantly, dyslipidaemia is responsive to changes in systemic inflammation, a recognized causal pathway of cardiovascular and cerebrovascular diseases [17–22]. Epidemiological evidence has indicated that blood lipids are associated with several inflammatory markers, such as cytokines and chemokines [17, 23]. Due to the inflammatory role of IgG *N*-glycosylation, together with its association with ageing [24], obesity [25], type 2 diabetes [26], hypertension [27, 28], ischemic stroke [29], Parkinson’s disease [30] and cancer [31], it can be speculated that blood lipids may be associated with the *N*-glycosylation of IgG.

In this study, we aimed to determine the association between the IgG *N*-glycome and blood lipids, including total cholesterol (TC), total triglycerides (TG), high-density lipoprotein (HDL), and low-density lipoprotein (LDL), and explored the use of IgG glycans as biomarkers to diagnose dyslipidaemia to further investigate the role of IgG glycosylation in dyslipidaemia.

## Methods

### Ethical approval

Written informed consent was obtained from each subject at the beginning of the study, and the study has been approved by the Ethics Committee of the Capital Medical University, Beijing, China. The ethics approval was given in compliance with the Declaration of Helsinki [32].

### Participant recruitment

An observational cross-sectional study was conducted during 2012, with a total of 913 participants of Chinese Han ancestry who were recruited from a community-based survey in Beijing [24, 27]. All participants were required to meet the following inclusion criteria: (1) age equal to or greater than 18 years; (2) no medication history during the previous 2 weeks; and (3) signed informed consent prior to participation. Individuals were excluded based on the following criteria: (1) pregnant or lactating women; (2) history of mental illness or infectious disease; and (3) history of malignant tumor, stroke or other cerebrovascular disease, congenital heart disease, acute myocardial infarction, liver disease, renal failure, chronic obstructive pulmonary disease, rheumatoid arthritis or other disease.

### Diagnosis of dyslipidaemia

According to the Chinese guidelines for the prevention and control of dyslipidaemia in adults [33], the participants were grouped into cases with TC ≥ 6.2 mmol/L (defined as hypercholesteremia), TG ≥ 2.3 mmol/L (defined as hypertriglyceridemia), HDL < 1.0 mmol/L (defined as the decreased HDL), or LDL ≥ 4.1 mmol/L (defined as the elevated LDL). The controls were those who did not have dyslipidaemia at the time of the study.

### Covariates

Each participant was required to complete a thorough health examination, which included anthropometric measurements and an analysis of the physical and chemical properties of a blood sample. Detailed information about subject recruitment and enrolment has been previously described [24, 27]. As in our previous study [24], 12 clinical traits (age, sex, body mass index (BMI), waist–hip ratio (WHR), systolic blood pressure (SBP), diastolic blood pressure (DBP), fasting blood triglycerides (FBG), resting heart rate (RHR), TC, TG, HDL and LDL) were assessed in the current study [24].

To obtain the anthropometric trait data, physical examinations and interviews were carried out by trained nurses and physicians. The BMI was calculated by the formula weight (in kilograms)/height^2^ (in metres squared). The SBP and DBP were measured twice on the right arm using a standard mercury sphygmomanometer after the subjects had rested for at least 10 min in a sitting position [34]. For the blood parameters (haematology and biochemical traits), fasting blood samples were collected in the morning after an overnight fast by venepuncture. All collected blood samples were processed within 8 h and blood plasma which was separated from whole blood was stored in freezers (− 80 °C) before the measurements. Haematology and biochemical parameters were measured by standard blood chemistry and haematology assays (Hitachi Automatic Analyzer, Model-7600, Tokyo, Japan), which were conducted at the Medical Laboratory of Beijing Xuanwu Hospital, Beijing.

### IgG *N*-glycan analysis

As previously reported, IgG was first isolated from human plasma [24, 35]. The IgG *N*-glycans were cleaved and analysed by hydrophilic interaction chromatography (HILIC)- ultra-performance liquid chromatography (UPLC) as previously described [35]. In brief, after washing and equilibrating the protein G monolithic plates, 50 µL of plasma was diluted 10× with binding buffer (1× phosphate-buffered saline, pH 7.4), applied to the protein G plates, and immediately washed. The IgGs were eluted with 1 mL of 0.1 M formic acid and immediately neutralized with 1 M ammonium bicarbonate. Next, the IgG *N*-glycans cleavage and labelling were performed. The released *N*-glycans were labelled with 2-aminobenzamide, a fluorescent dye used to visualize glycans by UPLC, by multistage mixing with 2-aminobenzamide, dimethyl sulfoxide, glacial acetic acid, and 2-picoline borane. Finally, the IgG *N*-glycans were analysed by HILIC–UPLC in 24 IgG glycan peaks (GPs). The glycan structures of the glycans per peak were previously reported, and a detailed description is shown in Additional file 1: Table S1 [35]. The normalization and batch correction of the UPLC data was detailed in a previous study [24, 35]. The total area normalization was applied where the amount of glycans in each peak was expressed as a percentage of the total integrated area. The calculation formula is shown in Additional file 1: Table S1.

Among the 24 initial GPs, GP3 was excluded as it did not pass quality control tests. An additional 54 derived traits were calculated for the remaining 23 initial glycans [35]. Of the derived traits, 34 were of interest and were included in the analysis describing the relative abundances of galactosylation, sialylation, bisecting GlcNAc and core fucosylation (Additional file 2: Table S2).

### Statistical analysis

The normal distribution of glycans was tested by the Kolmogorov–Smirnov test where *P *< 0.10 was considered to be statistically significant. Continuous variables underlying the normal distribution were represented as the mean ± standard deviation (SD), otherwise the medians (interquartile ranges) were used. The difference of continuous variables between two groups was tested using Student’s *t* test or the Wilcoxon rank-sum test. Categorical variables were represented as *n* (proportion), and the between-group differences were tested by the Chi square test. For the normally distributed variables, including age, BMI, WHR, SBP, DBP, FBG and RHR, *t*-tests were used to compare the differences between two groups. A generalized linear model (GLM) [36] was fitted to estimate the effect of blood lipids on glycosylation when adjusting for confounding factors, including age, sex, BMI, WHR, SBP, DBP, FBG and RHR, because most of the glycans (dependent variables) were not normally distributed. There were internal associations among the independent variables, which could have induced multi-collinearity in the statistical models. Therefore, principal component analysis (PCA) [37] was used to combine variables to principal components before the regression analysis. Moreover, these principal components were used in the multiple variables association analysis instead of the original variables. The internal associations among the independent variables were analysed using by the R package “corrplot” [38].

Before correlation and regression analysis, the z-score of normalized transformations for IgG *N*-glycans was applied to add the consistent comparability among IgG *N*-glycans. Canonical correlation analysis (CCA) [40] was used to determine two sets of variables of the initial glycan structures (x) and the blood lipids (y) and to find the overall correlation between the two sets of variables. The identified variables with a statistically significant impact on the canonical variables were judged by the canonical loadings. Generally, absolute values greater than 0.30 are considered to be significant loadings [39].

To explore IgG *N*-glycans as biomarkers to diagnose dyslipidaemia, 23 initial glycans were used as predictors. Logistic regression was performed to identify the 23 glycans that were respectively related to cases’ status after adjusting for the effect of age, sex, BMI, WHR, SBP, DBP, FBG and RHR. Considering the internal associations among these variables, we chose to correct sex, Prin.1 and Prin.2 instead of the original variables. The significant glycans were presented as the odds ratio (OR) with a 95% confidence interval (CI) and were graphed by a forest plot, which was generated using the R package “forestplot” [40]. Next, a classification model was established based on the significant glycans. Considering that there were internal associations in the glycans that might have induced multicollinearity in the model, the least absolute shrinkage and selection operator (LASSO) method was used to select glycans to reduce the dimension of data. The LASSO method was carried out by the R package “lars” [41]. The logistic regression model was then used to assess the discrimination of dyslipidaemia by combining the glycans left by the LASSO method. The classification model was only used to assess the ability of IgG *N*-glycans to be used as diagnostic biomarkers for disease; therefore, in the classification model there was no adjustment for the effect of age, sex, BMI, WHR, SBP, DBP, FBG and RHR. A receiver operating characteristic (ROC) curve was developed for the calculation of the area under the cure (AUC) with a 95% CI.

Data analysis was performed using SPSS Statistics version 21.0 for Windows (IBM Corp., Armonk, NY, USA), SAS software version 9.2 (SAS Institute, Chicago, IL, USA) and R version 3.3.2 (R Core Team 2016). All reported *P* values were two-sided, and *P *< 0.05 was considered statistically significant. Particularly, *P *< 0.05/57 (0.0009) was considered to be statistically significant in the analysis of glycans to correct for multiple comparisons.

## Results

### Description of clinical traits

Among the 913 recruited participants, 598 (64.50%) had a complete dataset of blood measurements and glycan traits and were included in the further analysis. In total, 8 clinical traits were described and compared between the dyslipidaemia and control groups (Table 1). Most of the traits (6 out of 8) were significantly different between the two groups (*P *< 0.05). The number of males and the values of age, BMI, WHR, FBG, and SBP in the dyslipidaemia group were significantly higher than those in the control group.

**Table 1:** Demographic and biochemical characteristics of the participants

Parameters	Total (n = 598)	Dyslipidaemia (n = 150)	Controls (n = 448)	P*
Number of male (%)	198 (33.11%)	76 (50.67%)	122 (27.23%)	< 0.001
Age (years)	47.35 ± 6.59	48.89 ± 7.02	46.84 ± 6.36	0.001
BMI (kg/m^2^)	24.59 ± 3.23	25.43 ± 3.65	24.28 ± 3.04	< 0.001
WHR	0.82 ± 0.07	0.85 ± 0.07	0.81 ± 0.07	< 0.001
FBG (mmol/L)	5.35 ± 0.83	5.58 ± 0.99	5.29 ± 0.77	< 0.001
SBP (mmHg)	117.69 ± 14.05	119.50 ± 13.84	117.26 ± 15.06	0.108
DBP (mmHg)	78.78 ± 10.46	82.06 ± 10.87	77.93 ± 10.20	< 0.001
RHR (beats/min)	76.09 ± 9.59	76.29 ± 8.91	76.13 ± 9.73	0.859

*BMI* body mass index, *WHR* waist–hip ratio, *FBG* fasting blood triglycerides, *SBP* systolic blood pressure, *DBP* diastolic blood pressure, *RHR* resting heart rate

* Statistically significant at significant level of 0.05

### Principal component analysis (PCA)

As shown in Additional file 3: Figure S1, the significant correlation coefficients in independent variables ranged from 0.08 to 0.89. PCA was used to combine a few variables to several principal components in order to reduce the multicollinearity of independent variables. The resulting Prin. 1 and Prin. 2 explained up to 54.78% of variance in SBP, DBP, FBG, RHR, age, BMI and WHR (Prin.1 = 0.773 × SBP + 0.783 × DBP + 0.575 × FBG + 0.106 × RHR + 0.352 × Age + 0.688 × BMI + 0.733 × WHR, Prin.2 = 0.361 × SBP + 0.364 × DBP − 0.176 × FBG + 0.739 × RHR − 0.370 × Age − 0.246 × BMI − 0.329 × WHR). The resulting Prin. 3 and Prin. 4 explained up to 85.65% of variance in TC, TG, HDL and LDL (Prin.3 = 0.985 × TC + 0.134 × TG + 0.444 × HDL + 0.923 × LDL, Prin.4 = 0.133 × TC + 0.889 × TG − 0.755 × HDL + 0.092 × LDL). Therefore, in the analysis of the association between IgG *N*-glycans and blood lipids, the principal components of blood lipids (Prin. 3 and Prin. 4) were used as independent variables instead of blood lipids, and they were adjusted for confounding factors (sex, Prin. 1 and Prin. 2).

### The association of IgG *N*-glycans with blood lipids

The results of the normal distribution tests of glycans are shown in Additional file 4: Table S3. Of the 23 directly measured glycans, 16 were not abnormally distributed (*P *< 0.10). Therefore, a GLM was used to estimate the effect of the principal components of blood lipids (Prin. 3 and Prin. 4) on glycosylation, adjusting for confounding factors (sex, Prin. 1 and Prin. 2). In total, 2 GPs in the IgG *N*-glycans were significantly associated with blood lipids after adjusting for the effects of sex, age, BMI, WHR, FBG, RHR, SBP and DBP (*P *< 0.05/57). As shown in Table 2, TC, TG and LDL were positively correlated with GP6, while they were negatively correlated with GP18. In addition, HDL negatively correlated with GP6 but positively correlated with GP18. Furthermore, 9 initial traits and 7 derived traits in the IgG glycome were significantly associated with blood lipids after adjusting for the effects of sex, age, BMI, WHR, FBG, RHR, SBP and DBP (*P *< 0.05). Detailed information is shown in the supplemental content (Additional file 5: Table S4).

**Table 2:** Associations between IgG glycan and blood lipids

IgG glycans	Prin. 3		Prin. 4	
	β (95% CI)^a^	*P*	β (95% CI)^b^	*P*
Initial measurements				
GP2	0.046 (0.006 to 0.087)	0.023*	− 0.012 (− 0.055 to 0.032)	0.596
GP4	0.035 (0.014 to 0.056)	0.001*	− 0.015 (− 0.039 to 0.010)	0.237
GP5	0.035 (0.014 to 0.055)	0.001*	0.019 (− 0.006 to 0.044)	0.130
GP6	0.036 (0.015 to 0.057)	< 0.001**	− 0.010 (− 0.034 to 0.014)	0.395
GP11	0.020 (0.004 to 0.036)	0.013*	0.017 (− 0.001 to 0.035)	0.066
GP14	− 0.022 (− 0.037 to 0.006)	0.006*	0.004 (− 0.014 to 0.022)	0.642
GP18	− 0.033 (− 0.051 to 0.015)	< 0.001**	0.006 (− 0.015 to 0.026)	0.604
GP20	0.014 (− 0.015 to 0.042)	0.336	0.051 (0.018 to 0.084)	0.003*
GP21	0.024 (0.004 to 0.045)	0.020*	0.025 (0.001 to 0.049)	0.041*
Sialylation				
FGS/(FG + FGS)	− 0.014 (− 0.025 to 0.003)	0.013*	0.001 (− 0.011 to 0.014)	0.840
FGS/(F + FG + FGS)	− 0.023 (− 0.037 to 0.008)	0.003*	0.006 (− 0.012 to 0.023)	0.527
Bisecting GlcNAc				
FBS^total^/FS^total^	0.021 (0.002 to 0.041)	0.032*	− 0.008 (− 0.030 to 0.014)	0.498
FBS2/FS2	0.020 (0.001 to 0.038)	0.039*	0.005 (− 0.016 to 0.023)	0.660
FBS2/(FS2 + FBS2)	0.011 (0.001 to 0.020)	0.034*	0.002 (− 0.009 to 0.013)	0.740
Galactosylation				
G0^n^	0.029 (0.012 to 0.046)	0.001*	–0.013 (− 0.033 to 0.007)	0.212
G2^n^	− 0.026 (− 0.043 to 0.009)	0.003*	0.008 (− 0.013 to 0.027)	0.476

Prin.1 = 0.773 × SBP + 0.783 × DBP + 0.575 × FBG + 0.106 × RHR + 0.352 × Age + 0.688 × BMI + 0.733 × WHR

Prin.2 = 0.361 × SBP + 0.364 × DBP − 0.176 × FBG + 0.739 × RHR − 0.370 × Age − 0.246 × BMI − 0.329 × WHR

Prin.3 = 0.985 × TC + 0.134 × TG + 0.444 × HDL + 0.923 × LDL

Prin.4 = 0.133 × TC + 0.889 × TG-0.755 × HDL + 0.092 × LDL

*BMI* body mass index, *WHR* waist–hip ratio, *FBG* fasting blood glucose, *SBP* systolic blood pressure, *DBP* diastolic blood pressure, *TC* total cholesterol, *TG* total triglycerides, *HDL* high-density lipoprotein, *LDL* low-density lipoprotein, *RHR* resting heart rate

^a^Adjusted for the effects of sex, Prin.1, Prin.2 and Prin.4

^b^Adjusted for the effects of sex, Prin.1, Prin.2 and Prin.3

* Statistically significant associations between two variables are shown, *P *< 0.05

** Statistically significant associations between two variables are shown, *P *< 0.05/57 = 0.0009

### Multivariate analyses by canonical correlation analysis (CCA)

**Fig. 1:**
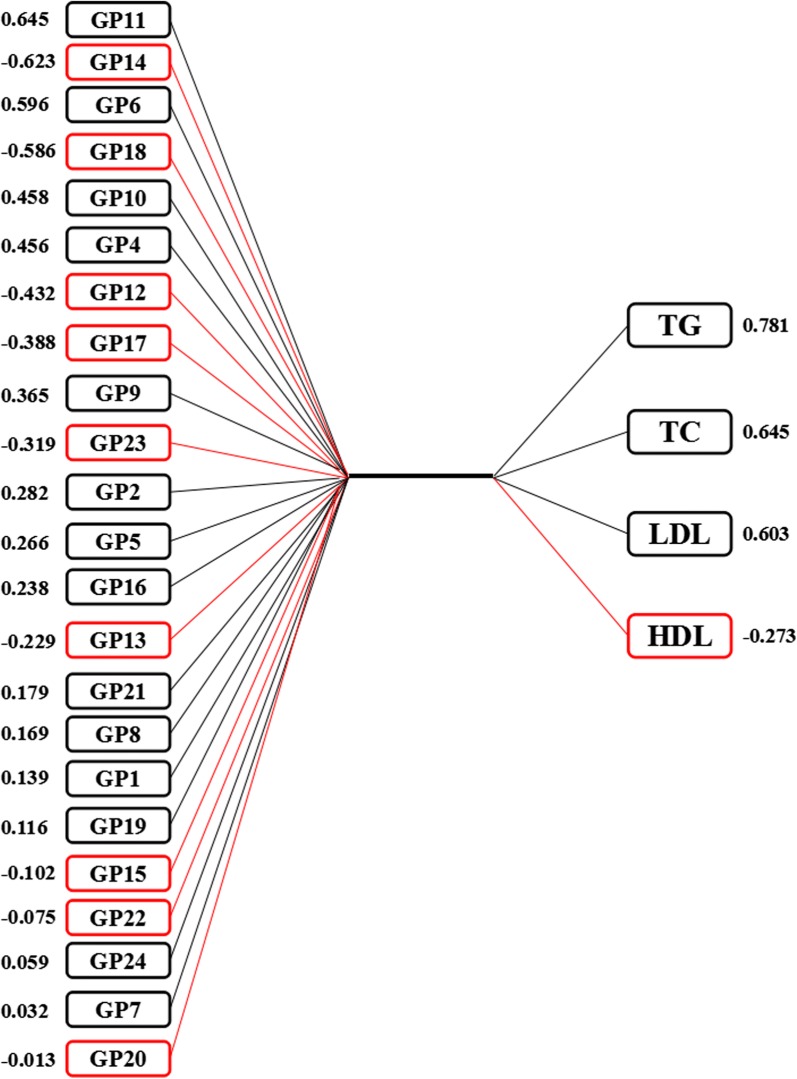
Canonical structures of the normalized IgG *N*-glycan and blood lipids in the first canonical set. The absolute value of canonical loadings greater than 0.30 was significant loadings. All of the variables are sorted by the absolute value of their canonical loadings. The positive relationships are represented in black boxes, while negative relationships are showed in red boxes. *TC* total cholesterol, *TG* total triglycerides, *HDL* high-density lipoprotein, *LDL* low-density lipoprotein

The results of CCA showed that there are 4 pairs of canonical variables, with canonical correlations of 0.390 (*F* = 2.44, *P* < 0.001), 0.324 (*F *= 1.88, *P* < 0.001), 0.240 (*F* = 1.39, *P* = 0.053) and 0.197 (*F* = 1.16, *P* = 0.282) for each successive pair by the CCA. The first and the second canonical sets were statistically significant, indicating that *N*-glycan structures were significantly correlated with the blood lipids. As shown in Fig. 1, 10 initial traits (GP4, GP6, GP9-12, GP14, GP17-18 and GP23) tended to be significantly associated with TC, TG and LDL levels in the first canonical set. In addition, the level of GP11 was strongly associated with canonical variables with a loading of 0.645, while the response variable with the highest canonical loading was 0.781 (TC).

### Classification of dyslipidaemia using IgG *N*-glycans

**Fig. 2:**
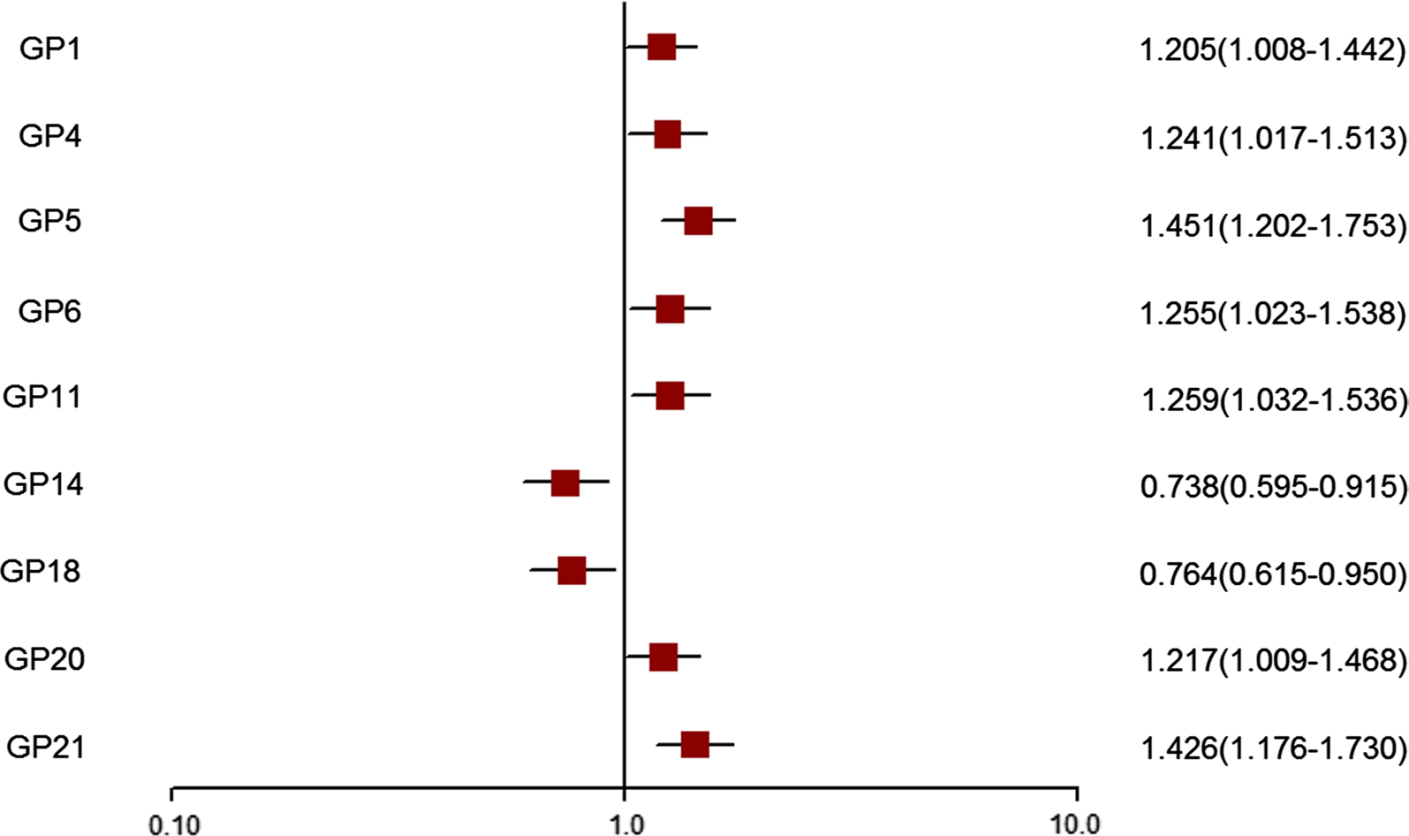
Odds ratios (OR) and 95% confidence intervals (95% CI) for the associations of the normalized glycan variables in dyslipidaemia vs controls (adjusted for sex, Prin.1 and Prin.2)

**Fig. 3:**
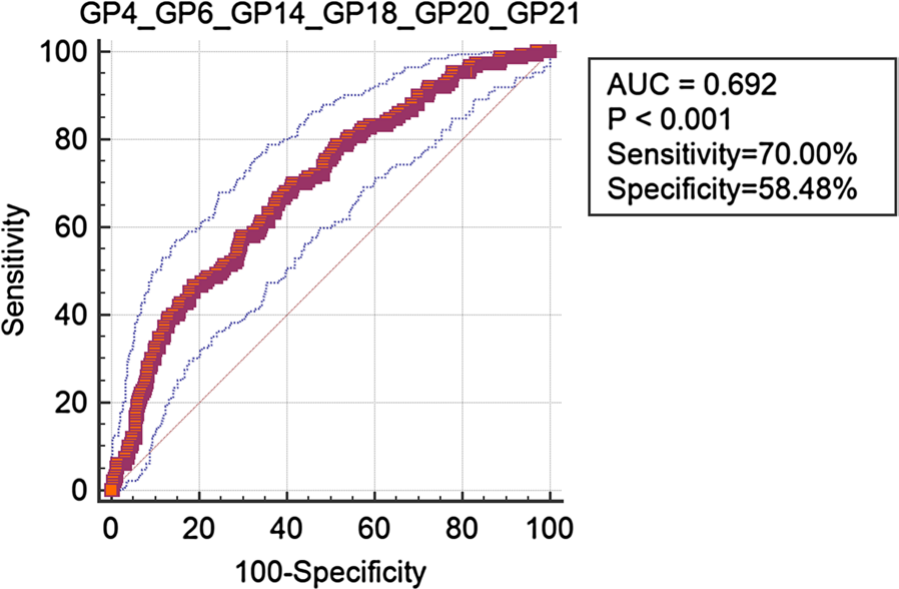
Receiver operating characteristic (ROC) curve analysis in regard to binary logistic regression in the prediction of dyslipidaemia. *AUC* area under the cure; GP4, GP6, GP14, GP18, GP20 and GP21 included in the final model

Among the 23 GPs, 9 GPs (GP1, GP4, GP5, GP6, GP11, GP14, GP18, GP20 and GP21) were found to be significantly different in participants with dyslipidaemia than in controls after adjusting for confounding factors (sex, Prin. 1 and Prin. 2) (Fig. 2 and Additional file 6: Table S5). As shown in Additional file 7: Figure S2, the significant correlation coefficients in independent variables ranged from 0.11 to 0.79. The LASSO method was used to select IgG *N*-glycans in order to reduce the dimension of data. The remaining glycans used by the LASSO method are listed in Additional file 8: Table S6. The classification model, incorporating significant IgG *N*-glycans, was able to distinguish dyslipidaemia from controls, and the AUC was 0.692 (95% CI 0.644–0.740) (Fig. 3).

## Discussion

Dyslipidaemia is one of the most important risk factors for atherosclerosis, which triggers the development of various cardiovascular and cerebrovascular diseases [13–16]. The increased TC, TG and LDL and the decreased HDL could be major public health problems; therefore, the identification of biomarkers that might provide new avenues for the prevention and treatment of dyslipidaemia is urgently needed. To our knowledge, this is the first study to investigate the association of IgG *N*-glycans with blood lipids and dyslipidaemia.

In the present study, the levels of TC, TG and LDL were positively associated with the levels of GP4 and GP6, while they were negatively correlated with the level of GP18. Furthermore, the results were consistent with those in CCA, in which *N*-glycan structures were related with blood lipids. GP4 and GP6 are agalactosylated glycans and share the derived trait G0^n^, which contains nearly the all glycans without galactose. In parallel, GP18 contains 2 galactoses, which was negatively correlated with blood lipids. In addition, GP6 that contains a bisecting GlcNAc was positively associated with blood lipids, while a significant negative correlation existed between blood lipids and GP18, in which 1 sialic acid was found. This is consistent with the results of the association between the derived traits in the IgG glycome and blood lipids. Our findings indicated that the associations between the blood lipids and IgG glycome were independently significant, with a negative association with diagalactosylation and sialylation and a positive association with bisecting agalactosylation and *N*-acetylgucosylation. Furthermore, the loss of galactose and sialic acid and the addition of a bisecting GlcNAc were consistently observed in patients with dyslipidaemia. The changes of IgG *N*-glycosylation in dyslipidaemia are consistent with the results of type 2 diabetes, hypertension and ischemic stroke [26, 27, 29, 42].

*N*-Glycans that are attached to the Fc portion of IgG are important modulators of IgG effector functions [5], and even slight changes in IgG glycosylation can direct pro- and anti-inflammatory actions of immunoglobulins [1, 4]. The decreased IgG galactosylation in rheumatoid arthritis was first reported nearly 32 years ago [43], and recently, the same type of change in IgG glycosylation has been reported in a number of autoimmune and inflammatory diseases [7, 9, 10, 12]. In addition, IgG glycosylation correlates with ageing, obesity, hypertension and cancer [24, 25, 27, 31]. It can be speculated that the loss of galactose is not disease specific but is a general phenomenon that is associated with reducing the anti-inflammatory function of circulating IgG. The addition of sialic acid dramatically changes the physiological role of IgGs, converting them from pro-inflammatory to anti-inflammatory agents [6, 44]. In the interactions between different functional elements of IgG glycosylation, it has been shown that the presence of bisecting GlcNAcs reduce galactosylated IgGs [6, 45, 46]. Accumulating evidence indicates that changes in IgG glycosylation mechanism could be part of the molecular mechanism leading to the promotion of inflammation [6, 8, 44, 47]; therefore, our results indicate inflammation as one of characteristics in dyslipidaemia that can increase the risk of developing other related disorders. This hypothesis is supported by our findings that IgG *N*-glycosylation is significantly associated with blood lipids and dyslipidaemia.

Previous studies have shown that the inflammatory role of IgG *N*-glycosylation is associated with the risk factors of dyslipidaemia [48] including aging and obesity [24, 25]. Inflammation, which is characteristic of aging and obesity, is a process associated with different disorders, such as dyslipidaemia [49]. Inflammatory disorders can lead to the activation of several signalling transduction pathways, inflammatory cytokine chemokine production and cell migration, all of which can influence lipid metabolism [49–52]. Interleukin 6 (IL-6) has been implicated in the pathogenesis of several immune-mediated diseases, and monoclonal antibodies directed against the IL-6 receptor have been developed to treat different inflammatory diseases [53]. *IL*-*6* and interleukin 6 signal transducer (*IL6ST*) have been identified as dyslipidaemia susceptibility loci [54, 55]. IL6ST has been identified to have the capability to regulate galactosylation of IgG in a genome-wide association study of IgG *N*-glycosylation [12]. According to the law of Mendelian randomization [56, 57], the genetic loci *IL6ST* is associated with IgG *N*-glycosylation and has an effect on dyslipidaemia, indicating that the change of galactosylation and inflammation is the intermediate phenotype between *IL6ST* and dyslipidaemia and is thus the causal factor of dyslipidaemia. However, this will need to be further explored and validated.

The aberrant glycosylation which induces inflammation may provide exciting insights into the pathogenesis of dyslipidaemia. However, causation is difficult to verify, and the observed changes may be the consequence rather than cause of the disease. Dyslipidaemia accompanied by adipocytes that can produce and secrete cytokines and adipokines [58] may thus affect the structural integrity of IgG glycans, where IgG plays a crucial role in the activation of complement, interacts with Fc receptors and affects antibody-dependent cell mediated cytotoxicity (ADCC) [5]. Dyslipidaemia, as a basic metabolic disease, may trigger changes in IgG glycosylation accompanied by inflammation that can lead to related diseases. Therefore, the casual effect between IgG glycosylation and dyslipidaemia remains unclear.

There are several potential limitations in this study that should be recognized. First, the study was performed with a relatively small sample population. Multiple correction was not used when we selected the initial glycans as diagnostic biomarkers, which may have led to false positive errors. However, to overcome this, the method of reducing the dimensions of data, including PCA and CCA, were applied to examine the association between level of *N*-glycans and blood lipids. The merit of PCA is that it explores all of the information from principal components, while the merit of CCA is to explore all of the information from two sets of variables; therefore, PCA and CCA help to solve the problem of multicollinearity that is induced by the similarities of the variables. Second, our study is a cross-sectional study, bringing the bias of diagnosis of dyslipidaemia cases. It lacks information regarding the time sequence of events; therefore, we cannot conclude the causal relationship of IgG glycosylation and dyslipidaemia. In addition, the present study is a pilot study to explore the association between *N*-glycans and blood lipids and dyslipidaemia. Further cohort studies or Mendelian randomization studies [57, 58] with larger sample sizes are needed to provide a more definite explanation about the relationships between *N*-glycan structures and dyslipidaemia.

## Conclusion

In conclusion, the present study showed a possible association between blood lipids and the observed loss of galactose and sialic acid, as well as the addition of bisecting GlcNAcs which might be related to the chronic inflammation accompanying the development of dyslipidaemia. Moreover, IgG *N*-glycosylation profiles may serve as potential biomarkers for dyslipidaemia, contributing to a move towards personalized medicine. Future studies focused on the mechanisms underlying the causal association of IgG glycosylation and blood lipids or dyslipidaemia are needed.

## Additional files

**Additional file 1: Table S1.** Structures of the initial IgG glycome.

**Additional file 2: Table S2.** The calculation formula of derived glycans.

**Additional file 3: Figure S1.** The correlation coefficients in independent variables Statistically significant associations between two variables are shown, while the insignificant correlation coefficients are blank in the boxes. The positive correlations are represented by blue color, while negative correlations are represented by red color. BMI: body mass index; WHR: waist–hip ratio; FBG: fasting blood triglycerides; SBP: systolic blood pressure; DBP: diastolic blood pressure; TC: total cholesterol; TG: triglyceride; HDL: high-density lipoprotein; LDL: low-density lipoprotein; RHR: resting heart rate.

**Additional file 4: Table S3.** Description of the IgG glycome.

**Additional file 5: Table S4.** Associations between IgG glycan and blood lipid.

**Additional file 6: Table S5.** The associations of the normalized glycan variables in dyslipidaemia vs controls.

**Additional file 7: Figure S2.** The correlation coefficients in glycans Statistically significant associations between two glycans are shown, while the insignificant correlation coefficients are blank in the boxes. The positive correlations are represented by blue color, while negative correlations are represented by red color.

**Additional file 8: Table S6.** The dimension reduction of significant glycans by LASSO method.

## Abbreviations

ADCC: antibody dependent cell-mediated cytotoxicity
AUC: area under the curve
BMI: body mass index
CCA: canonical correlation analysis
CI: confidence intervals
DBP: diastolic blood pressure
Fc: fragment crystallizable
FBG: fasting blood glucose
GLM: generalized linear model
GP: glycan peak
GPs: glycan peaks
HDL: high-density lipoprotein
HILIC: hydrophilic interaction chromatography
IgG: immunoglobulin G
IL-6: interleukin 6
IL6ST: interleukin 6 signal transducer
LASSO: least absolute shrinkage and selection operator
LDL: low-density lipoprotein
OR: odds ratio
PCA: principal component analysis
ROC: receiver operating characteristic
SBP: systolic blood pressure
SD: standard deviation
TC: total cholesterol
TG: triglycerides
UPLC: ultra performance liquid chromatography
WHO: World Health Organization
WHR: waist–hip ratio
