# Supplementary material for: The changes of immunoglobulin G N-glycosylation in blood lipids and dyslipidaemia
Source: J Transl Med. 2018 Aug 29;16:235. doi: 10.1186/s12967-018-1616-2 (PMC6114873; doi:10.1186/s12967-018-1616-2)
Supplement: Supplementary file 1 — Additional file 1: Table S1. Structures of the initial IgG glycome. [file 12967_2018_1616_MOESM1_ESM.docx]

Table S1 Structures of the initial IgG glycome

| Glycan peak | Peak composition | Formula/Normalization procedure |
| --- | --- | --- |
| GP1 | 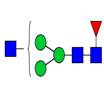 | GP1 / GP * 100 |
| GP2 | 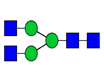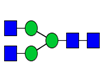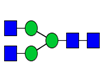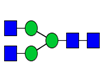 | GP2 / GP * 100 |
| GP4 | 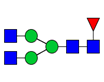 | GP4 / GP * 100 |
| GP5 | 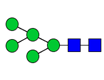 | GP5 / GP * 100 |
| GP6 | 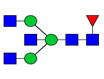 | GP6 / GP * 100 |
| GP7 | 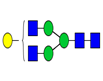 | GP7 / GP * 100 |
| GP8 | 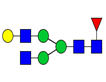 | GP8 / GP * 100 |
| GP9 | 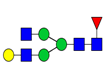 | GP9 / GP * 100 |
| GP10 | 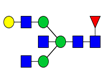 | GP10 / GP * 100 |
| GP11 | 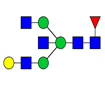 | GP11/ GP * 100 |
| GP12 | 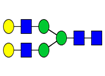 | GP12 / GP * 100 |
| GP13 | 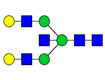 | GP13 / GP * 100 |
| GP14 | 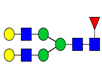 | GP14 / GP * 100 |
| GP15 | 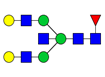 | GP15 / GP * 100 |
| GP16 | 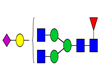 | GP16 / GP * 100 |
| GP17 | 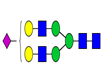 | GP17 / GP * 100 |
| GP18 | 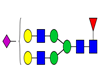 | GP18 / GP * 100 |
| GP19 | 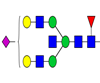 | GP19 / GP * 100 |
| GP20 | 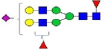 | GP20 / GP * 100 |
| GP21 | 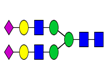 | GP21 / GP * 100 |
| GP22 | 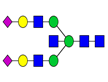 | GP22 / GP * 100 |
| GP23 | 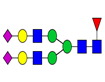 | GP23 / GP * 100 |
| GP24 | 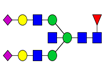 | GP24 / GP * 100 |

GP represents all the GPs. Blue squares represent bisecting GlcNAc, green circles represent mannose, red triangles represent fucose, yellow circles represent galactose, and purple diamonds represent sialic acid.
