# Supplementary material for: The changes of immunoglobulin G N-glycosylation in blood lipids and dyslipidaemia
Source: J Transl Med. 2018 Aug 29;16:235. doi: 10.1186/s12967-018-1616-2 (PMC6114873; doi:10.1186/s12967-018-1616-2)
Supplement: Supplementary file 2 — Additional file 2: Table S2. The calculation formula of derived glycans. [file 12967_2018_1616_MOESM2_ESM.docx]

Table S2. The calculation formula of derived glycans

| Glycans | Formula | Included in the study |
| --- | --- | --- |
| **Sialylation** | | |
| FGS/(FG+FGS) | SUM (GP16 + GP18 + GP23) / SUM (GP16 + GP18 + GP23 + GP8 + GP9 + GP14) * 100 | In |
| FBGS/(FBG+FBGS) | SUM (GP19 + GP24) / SUM (GP19 + GP24 + GP10 + GP11 + GP15) * 100 | In |
| FGS/(F+FG+FGS) | SUM (GP16 + GP18 + GP23) / SUM (GP16 + GP18 + GP23 + GP4 + GP8 + GP9 + GP14) * 100 | In |
| FBGS/(FB+FBG+FBGS) | SUM (GP19 + GP24) / SUM (GP19 + GP24 + GP6 + GP10 + GP11 + GP15) * 100 | In |
| FG1S1/(FG1+FG1S1) | GP16 / SUM (GP16 + GP8 + GP9) * 100 | In |
| FG2S1/(FG2+FG2S1+FG2S2) | GP18 / SUM (GP18 + GP14 + GP23) * 100 | In |
| FG2S2/(FG2+FG2S1+FG2S2) | GP23 / SUM (GP23 + GP14 + GP18) * 100 | In |
| FBG2S1/(FBG2+FBG2S1+FBG2S2) | GP19 / SUM (GP19 + GP15 + GP24) * 100 | In |
| FBG2S2/(FBG2+FBG2S1+FBG2S2) | GP24 / SUM (GP24 + GP15 + GP19) * 100 | In |
| F^total^S1/F^total^S2 | SUM (GP16 + GP18 + GP19) / SUM (GP23 + GP24) | In |
| FS1/FS2 | SUM (GP16 + GP18) / GP23 | In |
| FBS1/FBS2 | GP19 / GP24 | In |
| **Bisecting GlcNAc** | | |
| FBS^total^/FS^total^ | SUM (GP19 + GP24) / SUM (GP16 + GP18 + GP23) | In |
| FBS1/FS1 | GP19 / SUM (GP16 + GP18) | In |
| FBS2/FS2 | GP19 / SUM (GP16 + GP18 + GP19) | In |
| FBS1/(FS1+FBS1) | GP24 / GP23 | In |
| FBS2/(FS2+FBS2) | GP24 / SUM (GP23 + GP24) | In |
| **Neutral glycans** | | |
| GP1^n^ | GP1 / GP^n^* 100 |  |
| GP2^n^ | GP2 / GP^n^* 100 |  |
| GP4^n^ | GP4 / GP^n^* 100 |  |
| GP5^n^ | GP5 / GP^n^* 100 |  |
| GP6^n^ | GP6 / GP^n^* 100 |  |
| GP7^n^ | GP7 / GP^n^* 100 |  |
| GP8^n^ | GP8 / GP^n^* 100 |  |
| GP9^n^ | GP9 / GP^n^* 100 |  |
| GP10^n^ | GP10 / GP^n^* 100 |  |
| GP11^n^ | GP11 / GP^n^* 100 |  |
| GP12^n^ | GP12 / GP^n^* 100 |  |
| GP13^n^ | GP13 / GP^n^* 100 |  |
| GP14^n^ | GP14 / GP^n^* 100 |  |
| GP15^n^ | GP15 / GP^n^* 100 |  |
| **Galactosylation** | | |
| G0^n^ | SUM (GP1^n^: GP4^n^ + GP6^n^) | In |
| G1^n^ | SUM (GP7^n^: GP11^n^) | In |
| G2^n^ | SUM (GP12^n^: GP15^n^) | In |
| **Core fucosylation and bisecting GlcNAc** | | |
| F^ntotal^ | SUM (GP1^n^+ GP4^n^+ GP6^n^+ GP8^n^+ GP9^n^+ GP10^n^+ GP11^n^+ GP14^n^+P15^n^) | In |
| FG0^ntotal^/G0^n^ | SUM (GP1^n^+ GP4^n^+ GP6^n^) / G0^n^ * 100 | In |
| FG1^ntotal^/G1^n^ | SUM (GP8^n^+ GP9^n^+ GP10^n^+ GP11^n^) / G1^n^ * 100 | In |
| FG2^ntotal^/G2^n^ | SUM (GP14^n^+ GP15) / G2^n^ * 100 | In |
| F^n^ | SUM (GP1^n^+ GP4^n^+ GP8^n^+ GP9^n^+ GP14^n^) | In |
| FG0^n^/G0^n^ | SUM (GP1^n^+ GP4^n^) / G0^n^ * 100 | In |
| FG1^n^/G1^n^ | SUM (GP8^n^+ GP9^n^) / G1^n^ * 100 | In |
| FG2^n^/G2^n^ | GP14^n^ / G2^n^ * 100 | In |
| FB^n^ | SUM (GP6^n^ + GP10^n^ + GP11^n^ + GP15^n^) | In |
| FBG0^n^/G0^n^ | GP6^n^/ G0^n^ * 100 | In |
| FBG1^n^/G1^n^ | SUM (GP10^n^ + GP11^n^) / G1^n^ * 100 | In |
| FBG2^n^/G2^n^ | GP15^n^ / G2^n^ * 100 | In |
| FB^n^/F^n^ | FB^n^ / F^n^ * 100 | In |
| F^n^/(B^n^+FB^n^) | FB^n^ / F^n^ total * 100 |  |
| B^n^/(F^n^+FB^n^) | F^n^ / (GP13^n^ + FB^n^) |  |
| FBG2^n^/FG2^n^ | GP13^n^ / (F^n^+ FB^n^) * 1000 |  |
| FG2^n^/(BG2^n^+FBG2^n^) | GP15^n^ / GP14^n^ |  |
| BG2^n^/(FG2^n^+FBG2^n^) | GP15^n^ / (GP14^n^ + GP15^n^) * 100 |  |
| FB^n^/F^ntotal^ | GP14^n^ / (GP13^n^ + GP15^n^) | In |
| FBG2^n^/(FG2^n^+FBG2^n^) | GP13^n^ / (GP14^n^ + GP15^n^) * 1000 |  |
