# Supplementary material for: The changes of immunoglobulin G N-glycosylation in blood lipids and dyslipidaemia
Source: J Transl Med. 2018 Aug 29;16:235. doi: 10.1186/s12967-018-1616-2 (PMC6114873; doi:10.1186/s12967-018-1616-2)
Supplement: Supplementary file 3 — Additional file 3: Figure S1. The correlation coefficients in independent variables Statistically significant associations between two variables are shown, while the insignificant correlation coefficients are blank in the boxes. The positive correlations are represented by blue color, while negative correlations are represented by red color. BMI: body mass index; WHR: waist–hip ratio; FBG: fasting blood triglycerides; SBP: systolic blood pressure; DBP: diastolic blood pressure; TC: total cholesterol; TG: triglyceride; HDL: high-density lipoprotein; LDL: low-density lipoprotein; RHR: resting heart rate. [file 12967_2018_1616_MOESM3_ESM.docx]

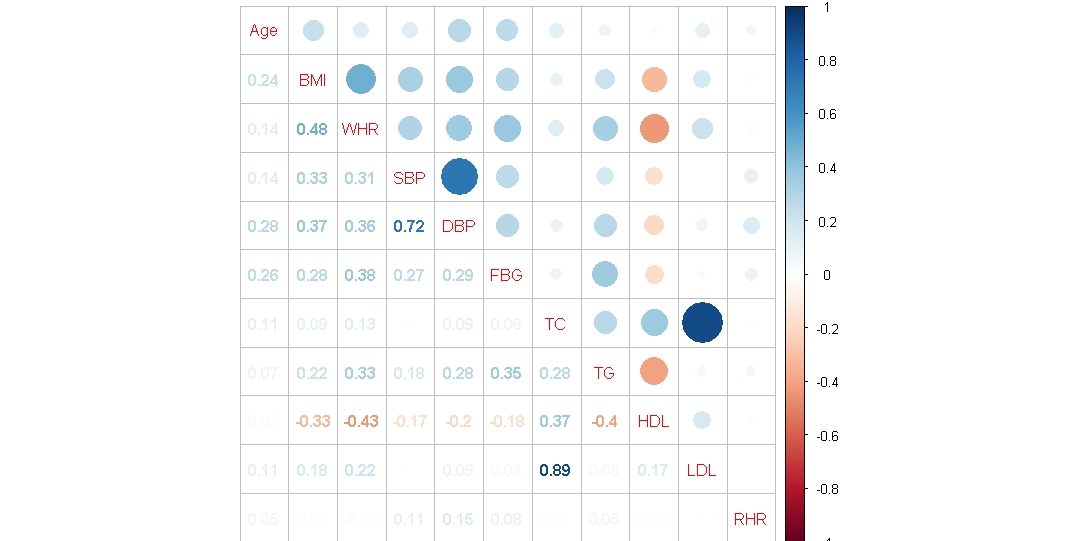


**Figure S1 The correlation coefficients in independent variables**

Statistically significant associations between two variables are shown, while the insignificant correlation coefficients are blank in the boxes. The positive correlations are represented by blue color, while negative correlations are represented by red color. BMI: body mass index; WHR: waist-hip ratio; FBG: fasting blood triglycerides; SBP: systolic blood pressure; DBP: diastolic blood pressure; TC: total cholesterol; TG: triglyceride; HDL: high-density lipoprotein; LDL: low-density lipoprotein; RHR: resting heart rate.
