# Supplementary material for: The changes of immunoglobulin G N-glycosylation in blood lipids and dyslipidaemia
Source: J Transl Med. 2018 Aug 29;16:235. doi: 10.1186/s12967-018-1616-2 (PMC6114873; doi:10.1186/s12967-018-1616-2)
Supplement: Supplementary file 4 — Additional file 4: Table S3. Description of the IgG glycome. [file 12967_2018_1616_MOESM4_ESM.docx]

Table S3 Description of the initial IgG glycome

| Glycan peak | Range  (min-max) | Z | *P** |
| --- | --- | --- | --- |
| GP1 | 0.02-0.47 | 4.457 | <0.001 |
| GP2 | 0.05-3.27 | 3.041 | <0.001 |
| GP4 | 4.68-38.26 | 1.336 | 0.056 |
| GP5 | 0.07-0.71 | 3.026 | <0.001 |
| GP6 | 1.30-8.91 | 2.256 | <0.001 |
| GP7 | 0.15-2.33 | 2.194 | <0.001 |
| GP8 | 13.94-25.64 | 0.546 | 0.926 |
| GP9 | 4.52-14.06 | 0.546 | 0.927 |
| GP10 | 2.11-10.54 | 1.353 | 0.051 |
| GP11 | 0.32-1.14 | 1.336 | 0.056 |
| GP12 | 0.26-3.58 | 2.214 | <0.001 |
| GP13 | 0.16-1.31 | 2.096 | <0.001 |
| GP14 | 5.64-29.93 | 0.578 | 0.892 |
| GP15 | 0.65-3.33 | 1.193 | 0.116 |
| GP16 | 1.42-4.61 | 0.762 | 0.607 |
| GP17 | 0.48-2.57 | 2.132 | <0.001 |
| GP18 | 5.52-24.95 | 1.143 | 0.147 |
| GP19 | 1.10-3.29 | 2.239 | <0.001 |
| GP20 | 0.12-1.25 | 2.543 | <0.001 |
| GP21 | 0.02-0.63 | 1.505 | 0.022 |
| GP22 | 0.50-4.13 | 3.943 | <0.001 |
| GP23 | 0.50-4.13 | 1.513 | 0.021 |
| GP24 | 0.53-3.91 | 0.865 | 0.443 |

* Normality distributions of glycans were tested by the Kolmogorov-Smirnov tests, and of which *P*< 0.10 was considered statistically significant.
