# Supplementary material for: The changes of immunoglobulin G N-glycosylation in blood lipids and dyslipidaemia
Source: J Transl Med. 2018 Aug 29;16:235. doi: 10.1186/s12967-018-1616-2 (PMC6114873; doi:10.1186/s12967-018-1616-2)
Supplement: Supplementary file 5 — Additional file 5: Table S4. Associations between IgG glycan and blood lipid. [file 12967_2018_1616_MOESM5_ESM.docx]

Table S4 Associations between IgG glycan and blood lipid

| **IgG glycan** | **Prin. 3** | | **Prin. 4** | | | | | |
| --- | --- | --- | --- | --- | --- | --- | --- | --- |
|  | β(95%CI^)#^ | *P* | β(95%CI)^&^ | | | *P* | | |
| **Initial measurements** | | | | | | | | |
| GP1 | 0.037(-0.005-0.079) | 0.088 | -0.002(-0.052-0.048) | | | 0.934 | | |
| GP2 | **0.046(0.006-0.087)** | **0.023***** | -0.012(-0.055-0.032) | | | 0.596 | | |
| GP4 | **0.035(0.014-0.056)** | **0.001***** | -0.015(-0.039-0.010) | | | 0.237 | | |
| GP5 | **0.035(0.014-0.055)** | **0.001***** | 0.019(-0.006-0.044) | | | 0.130 | | |
| GP6 | **0.036(0.015-0.057)** | **<0.001****** | -0.010(-0.034-0.014) | | | 0.395 | | |
| GP7 | 0.010(-0.023-0.043) | 0.546 | 0.004(-0.033-0.040) | | | 0.835 | | |
| GP8 | 0.002(-0.006-0.009) | 0.622 | -0.001(-0.010-0.007) | | | 0.780 | | |
| GP9 | 0.012(0.001-0.024) | 0.048 | 0.004(-0.010-0.017) | | | 0.597 | | |
| GP10 | 0.007(-0.009-0.023) | 0.414 | 0.009(-0.009-0.027) | | | 0.335 | | |
| GP11 | **0.020(0.004-0.036)** | **0.013***** | 0.017(-0.001-0.035) | | | 0.066 | | |
| GP12 | -0.029(-0.064-0.005) | 0.095 | 0.003(-0.036-0.042) | | | 0.870 | | |
| GP13 | -0.001(-0.021-0.021) | 0.992 | 0.011(-0.013-0.036) | | | 0.367 | | |
| GP14 | **-0.022(-0.037--0.006)** | **0.006***** | 0.004(-0.014-0.022) | | | 0.642 | | |
| GP15 | -0.012(-0.030-0.006) | 0.189 | 0.019(-0.002-0.039) | | | 0.076 | | |
| GP16 | 0.006(-0.008-0.019) | 0.421 | 0.003(-0.012-0.018) | | | 0.690 | | |
| GP17 | -0.008(-0.028-0.012) | 0.434 | -0.004(-0.026-0.019) | | | 0.765 | | |
| GP18 | **-0.033(-0.051--0.015)** | **<0.001****** | 0.006(-0.015-0.026) | | | 0.604 | | |
| GP19 | -0.005(-0.020-0.010) | 0.513 | 0.001(-0.016-0.017) | | | 0.950 | | |
| GP20 | 0.014(-0.015-0.042) | 0.336 | **0.051(0.018-0.084)** | | | **0.003***** | | |
| GP21 | **0.024(0.004-0.045)** | **0.020***** | **0.025(0.001-0.049)** | | | **0.041***** | | |
| GP22 | -0.009(-0.045-0.026) | 0.591 | 0.039(-0.001-0.079) | | | 0.058 | | |
| GP23 | -0.024(-0.053-0.005) | 0.104 | -0.002(-0.035-0.032) | | | 0.916 | | |
| GP24 | -0.004(-0.027-0.019) | 0.726 | -0.002(-0.026-0.024) | | | 0.894 | | |
| **Sialylation** | | | | | | | | |
| FGS/(FG+FGS) | **-0.014(-0.025--0.003)** | **0.013***** | 0.001(-0.011-0.014) | | | 0.840 | | |
| FBGS/(FBG+FBGS) | -0.003(-0.018-0.012) | 0.673 | -0.009(-0.026-0.009) | | | 0.332 | | |
| FGS/(F+FG+FGS) | **-0.023(-0.037--0.008)** | **0.003***** | 0.006(-0.012-0.023) | | | 0.527 | | |
| FBGS/(FB+FBG+FBGS) | -0.012(-0.029-0.005) | 0.154 | -0.003(-0.022-0.017) | | | 0.776 | | |
| FG1S1/(FG1+FG1S1) | 0.001(-0.014-0.014) | 0.972 | 0.003(-0.013-0.019) | | | 0.756 | | |
| FG2S1/(FG2+FG2S1+FG2S2) | -0.005(-0.012-0.001) | 0.119 | -0.001(-0.008-0.007) | | | 0.935 | | |
| FG2S2/(FG2+FG2S1+FG2S2) | 0.010(-0.015-0.036) | 0.426 | -0.012(-0.0041-0.018) | | | 0.437 | | |
| FBG2S1/(FBG2+FBG2S1+FBG2S2) | 0.003(-0.006-0.012) | 0.499 | -0.005(-0.015-0.005) | | | 0.338 | | |
| FBG2S2/(FBG2+FBG2S1+FBG2S2) | 0.004(-0.013-0.020) | 0.641 | -0.008(-0.027-0.011) | | | 0.402 | | |
| F^total^S1/F^total^S2 | -0.005(-0.026-0.017) | 0.665 | 0.003(-0.023-0.028) | | | 0.840 | | |
| FS1/FS2 | 0.004(-0.021-0.028) | 0.765 | 0.008(-0.020-0.037) | | | 0.556 | | |
| FBS1/FBS2 | -0.001(-0.021-0.020) | 0.982 | -0.001(-0.025-0.022) | | | 0.923 | | |
| **Bisecting GlcNAc** | | | | | | | | |
| FBS^totaL^/FS^total^ | **0.021(0.002-0.041)** | **0.032***** | -0.008(-0.030-0.014) | | | 0.498 | | |
| FBS1/FS1 | 0.021(-0.001-0.043) | 0.058 | -0.007(-0.032-0.018) | | | 0.575 | | |
| FBS1/(FS1+FBS1) | 0.019(-0.001-0.038) | 0.057 | -0.005(-0.027-0.017) | | | 0.651 | | |
| FBS2/FS2 | **0.020(0.001-0.038)** | **0.039***** | 0.005(-0.016-0.023) | | | 0.660 | | |
| FBS2/(FS2+FBS2) | **0.011(0.001-0.020)** | **0.034***** | 0.002(-0.009-0.013) | | | 0.740 | | |
| **Galactosylation** | | | | | | |  |  |
| G0^n^ | **0.029(0.012-0.046)** | **0.001***** | -0.013(-0.033-0.007) | | | 0.212 | | |
| G1^n^ | 0.001(-0.003-0.005) | 0.752 | 0.004(-0.001-0.008) | | | 0.130 | | |
| G2^n^ | **-0.026(-0.043--0.009)** | **0.003***** | 0.008(-0.013-0.027) | | | 0.476 | | |
| **Core fucosylation and bisecting GlcNAc** | | | | | | | |  |
| F^n total^ | 0.0003(-0.001-0.001) | 0.597 | | -0.0002(-0.001-0.001) | 0.763 | | |  |
| FG0^n total^/G0^n^ | -0.0003(-0.001-0.0005) | 0.461 | | -0.0004(-0.001-0.0005) | 0.431 | | |  |
| FG1^n total^/G1^n^ | -0.0001(-0.001-0.001) | 0.888 | | -0.0001(-0.001-0.001) | 0.829 | | |  |
| FG2^n total^ /G2^n^ | 0.0003(-0.001-0.002) | 0.739 | | 0.0003(-0.002-0.002) | 0.792 | | |  |
| F^n^ | -0.001(-0.004-0.002) | 0.436 | | 0.001(-0.005-0.002) | 0.450 | | |  |
| FG0^n^/G0^n^ | -0.001(-0.005-0.003) | 0.654 | | -0.001(-0.006-0.003) | 0.544 | | |  |
| FG1^n^/G1^n^ | -0.001(-0.004-0.003) | 0.740 | | -0.002(-0.005-0.002) | 0.337 | | |  |
| FG2^n^/G2^n^ | -0.001(-0.003-0.002) | 0.675 | | -0.001(-0.004-0.002) | 0.497 | | |  |
| FB^n^ | 0.008(-0.005-0.021) | 0.226 | | 0.006(-0.010-0.021) | 0.466 | | |  |
| FBG0^n^/G0^n^ | 0.002(-0.012-0.017) | 0.759 | | 0.004(-0.012-0.020) | 0.653 | | |  |
| FBG1^n^/G1^n^ | 0.002(-0.012-0.017) | 0.764 | | 0.008(-0.009-0.025) | 0.338 | | |  |
| FBG2^n^/G2^n^ | 0.008(-0.006-0.022) | 0.241 | | 0.012(-0.004-0.028) | 0.138 | | |  |
| FB^n^/F^n^ | 0.010(-0.006-0.026) | 0.232 | | 0.007(-0.011-0.025) | 0.463 | | |  |
| FB^n^/F^n total^ | 0.008(-0.006-0.021) | 0.249 | | 0.006(-0.010-0.02) | 0.463 | | |  |

# Adjusted for the effects of sex, Prin.1, Prin.2 and Prin.4.

& Adjusted for the effects of sex, Prin.1, Prin.2 and Prin.3.

* Statistically significant associations between two variables are shown，*P*<0.05.

**Statistically significant associations between two variables are shown，*P*<0.05/57=0.0009

Prin.1=0.773×SBP+0.783×DBP+0.575×FBG+0.106×RHR+0.352×Age+0.688×BMI+0.733×WHR

Prin.2=0.361×SBP+0.364×DBP-0.176×FBG+0.739×RHR-0.370×Age-0.246×BMI-0.329×WHR

Prin.3=0.985×TC+0.134×TG+0.444×HDL+0.923×LDL

Prin.4=0.133×TC+0.889×TG-0.755×HDL+0.092×LDL

BMI: body mass index; WHR: waist-hip rate; FBG: fasting blood glucose; SBP: systolic blood pressure; DBP: diastolic blood pressure; TC: total cholesterol; TG: total triglycerides; HDL: high density lipoprotein; LDL: low density lipoprotein; RHR: resting heart rate.
