# Supplementary material for: The changes of immunoglobulin G N-glycosylation in blood lipids and dyslipidaemia
Source: J Transl Med. 2018 Aug 29;16:235. doi: 10.1186/s12967-018-1616-2 (PMC6114873; doi:10.1186/s12967-018-1616-2)
Supplement: Supplementary file 6 — Additional file 6: Table S5. The associations of the normalized glycan variables in dyslipidaemia vs controls. [file 12967_2018_1616_MOESM6_ESM.docx]

Table S5 The associations of the normalized glycan variables in dyslipidaemia vs controls

| Glycan peak | OR(95% CI) | *P* | AOR(95% CI)^#^ | *P* |
| --- | --- | --- | --- | --- |
| GP1 | 1.249(1.051-1.484) | 0.012* | 1.205(1.008-1.442) | 0.041* |
| GP2 | 1.288(1.067-1.555) | 0.008* | 1.111(0.922-1.339) | 0.269 |
| GP4 | 1.443(1.201-1.733) | <0.001** | 1.241(1.017-1.513) | 0.033* |
| GP5 | 1.497(1.249-1.795) | <0.001** | 1.451(1.202-1.753) | <0.001** |
| GP6 | 1.523(1.270-1.827) | <0.001** | 1.255(1.023-1.538) | 0.029* |
| GP7 | 1.152(0.963-1.378) | 0.121 | 1.110(0.922-1.337) | 0.270 |
| GP8 | 1.062(0.883-1.277) | 0.526 | 0.999(0.824-1.211) | 0.993 |
| GP9 | 1.164(0.966-1.402) | 0.110 | 1.080(0.889-1.312) | 0.440 |
| GP10 | 1.208(1.007-1.448) | 0.042* | 1.046(0.864-1.266) | 0.648 |
| GP11 | 1.459(1.210-1.758) | <0.001** | 1.259(1.032-1.536) | 0.023* |
| GP12 | 0.807(0.661-0.986) | 0.036* | 0.908(0.739-1.117) | 0.362 |
| GP13 | 0.987(0.819-1.189) | 0.890 | 1.122(0.922-1.365) | 0.251 |
| GP14 | 0.613(0.504-0.744) | <0.001** | 0.738(0.595-0.915) | 0.006* |
| GP15 | 0.861(0.713-1.039) | 0.119 | 0.925(0.760-1.126) | 0.437 |
| GP16 | 1.118(0.929-1.345) | 0.239 | 1.075(0.888-0.1302) | 0.457 |
| GP17 | 0.879(0.724-1.066) | 0.190 | 0.991(0.809-1.214) | 0.931 |
| GP18 | 0.632(0.516-0.774) | <0.001** | 0.764(0.615-0.950) | 0.015* |
| GP19 | 1.109(0.924-1.331) | 0.265 | 1.033(0.854-1.251) | 0.738 |
| GP20 | 1.099(0.920-1.314) | 0.299 | 1.217(1.009-1.468) | 0.040* |
| GP21 | 1.366(1.137-1.641) | 0.001 | 1.426(1.176-1.730) | <0.001** |
| GP22 | 1.014(0.844-1.218) | 0.881 | 1.055(0.872-1.275) | 0.584 |
| GP23 | 0.881(0.729-1.065) | 0.190 | 0.954(0.784-1.162) | 0.640 |
| GP24 | 1.062(0.883-1.277) | 0.523 | 1.010(0.834-1.222) | 0.921 |

# Adjusted for sex, Prin.1 and Prin.2

* Statistically significant associations between two variables are shown，*P*<0.05.

**Statistically significant associations between two variables are shown，*P*<0.05/57=0.0009

Prin.1=0.773×SBP+0.783×DBP+0.575×FBG+0.106×RHR+0.352×Age+0.688×BMI+0.733×WHR

Prin.2=0.361×SBP+0.364×DBP-0.176×FBG+0.739×RHR-0.370×Age-0.246×BMI-0.329×WHR
