# Supplementary material for: The changes of immunoglobulin G N-glycosylation in blood lipids and dyslipidaemia
Source: J Transl Med. 2018 Aug 29;16:235. doi: 10.1186/s12967-018-1616-2 (PMC6114873; doi:10.1186/s12967-018-1616-2)
Supplement: Supplementary file 7 — Additional file 7: Figure S2. The correlation coefficients in glycans Statistically significant associations between two glycans are shown, while the insignificant correlation coefficients are blank in the boxes. The positive correlations are represented by blue color, while negative correlations are represented by red color. [file 12967_2018_1616_MOESM7_ESM.docx]

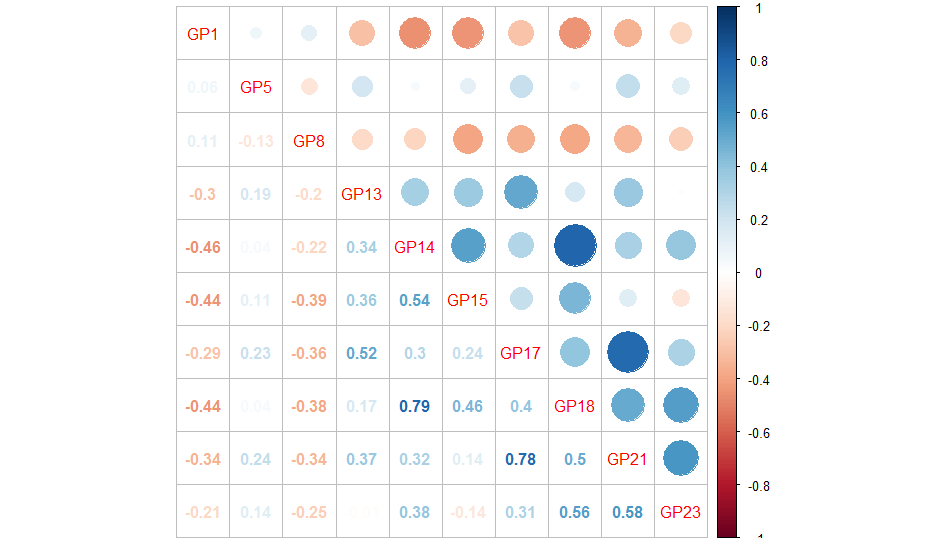


**Figure S2 The correlation coefficients in glycans**

Statistically significant associations between two glycans are shown, while the insignificant correlation coefficients are blank in the boxes. The positive correlations are represented by blue color, while negative correlations are represented by red color.
