# Supplementary material for: The changes of immunoglobulin G N-glycosylation in blood lipids and dyslipidaemia
Source: J Transl Med. 2018 Aug 29;16:235. doi: 10.1186/s12967-018-1616-2 (PMC6114873; doi:10.1186/s12967-018-1616-2)
Supplement: Supplementary file 8 — Additional file 8: Table S6. The dimension reduction of significant glycans by LASSO method. [file 12967_2018_1616_MOESM8_ESM.docx]

Table S6 The dimension reduction of significant glycans by LASSO method

| Glycans | Coefficients |
| --- | --- |
| GP1 | / |
| GP4 | -0.021 |
| GP5 | / |
| GP6 | 0.121 |
| GP11 | / |
| GP14 | -0.076 |
| GP18 | -0.109 |
| GP20 | 0.330 |
| GP21 | 1.799 |

/ The glycans are not left by the LASSO method.
